# Psychological Effects of Hemodialysis on Patients with Renal Failure: A Cross-Sectional Study

**DOI:** 10.3390/jcm14207136

**Published:** 2025-10-10

**Authors:** Abdulaziz M. Bakhsh, Waleed H. Mahallawi

**Affiliations:** 1 General and Specialized Surgery Department, College of Medicine, Taibah University, Madinah 42353, Saudi Arabia; bakhshaziz@gmail.com; 2 Clinical Laboratory Sciences Department, College of Applied Medical Sciences, Taibah University, Madinah 42353, Saudi Arabia; 3 Health and Life Research Center, Taibah University, Madinah 42353, Saudi Arabia

**Keywords:** psychological effects, hemodialysis, renal failure, anxiety, depression, quality of life, resilience, Saudi Arabia

## Abstract

**Background**: End-stage renal disease (ESRD) presents a substantial and growing global health challenge, where hemodialysis serves as an essential life-sustaining therapy for countless individuals. Despite its physiological necessity, the demanding treatment regimen can profoundly impact mental health and overall well-being, though gender-specific data and correlates within the Saudi population remain insufficiently explored. **Methods**: This cross-sectional study aimed to investigate this gap by assessing the prevalence of anxiety and depression, evaluating health-related quality of life (HRQoL), and analyzing associations with gender and treatment duration in a cohort of 250 hemodialysis patients from multiple centers in Madinah, Saudi Arabia. Validated instruments, namely, the Hospital Anxiety and Depression Scale (HADS) and the 36-Item Short Form Health Survey (SF-36), were employed. **Results**: The findings revealed a significant psychological burden, with 38% of patients exhibiting anxiety and 32% depression, with females disproportionately affected. HRQoL scores were severely diminished across all domains compared to healthy population norms. Furthermore, a longer dialysis vintage demonstrated a significant positive correlation with worsening psychological scores and a decline in physical HRQoL. **Conclusions**: These results underscore the critical need for a paradigm shift in standard care, advocating for the systematic integration of routine mental health screenings and the development of tailored, gender-sensitive psychosocial interventions to mitigate this considerable burden.

## 1. Introduction

Chronic kidney disease (CKD) represents a pervasive and progressive global health crisis, affecting approximately 10% of the world’s population and culminating in end-stage renal disease (ESRD) for over two million individuals who depend on renal replacement therapy for survival [1]. Hemodialysis (HD), while technologically advanced and physiologically vital, is far from a curative solution. It enforces a relentless and demanding regimen of thrice-weekly sessions, stringent dietary restrictions, and meticulous fluid limitations, which collectively orchestrate a fundamental disruption to a patient’s life, often precipitating a profound loss of personal and professional autonomy [2]. The psychological sequelae of this protracted and dependent state are severe and extensively documented within the literature. Epidemiological studies consistently report that between 20% and 50% of hemodialysis patients experience clinically significant levels of anxiety or depression, which are not merely comorbidities but powerful predictors of detrimental outcomes, including poor treatment adherence, elevated hospitalization rates, and increased all-cause mortality [3,4]. Alarmingly, despite the robust evidence linking psychological health to survival, mental health screening and intervention continue to be critically underprioritized and systematically overlooked in routine nephrology care, creating a chasm between patient needs and clinical practice [5].

The distribution of this psychological burden is not homogenous, and gender-specific disparities introduce a layer of complexity to patient outcomes. Women on hemodialysis frequently navigate a dual burden, managing the exigencies of their own life-limiting illness while simultaneously striving to fulfill entrenched societal and familial caregiving roles, a conflict that can significantly exacerbate their emotional strain and sense of guilt [6]. Conversely, men may systematically underreport psychological symptoms due to cultural stigma and constructs of masculinity that discourage the expression of emotional vulnerability, effectively masking their distress and precluding access to necessary support [7]. Beyond the dimensions of anxiety and depression, Health-related quality of life (HRQoL), a critical multi-dimensional indicator encompassing physical, mental, and social well-being, is frequently and severely compromised in this population. Yet, comprehensive assessments that integratively analyze both physical and mental health domains within specific cultural and regional contexts, such as Saudi Arabia, remain relatively scarce and constitute a significant gap in the extant literature [8].

Emerging research has begun to illuminate potential protective factors that may buffer against this distress. The concept of psychological resilience—defined as the capacity to adapt flexibly, withstand adversity, and recover from challenges—has gained prominence as a key moderating variable [9]. Growing evidence suggests that higher levels of resilience may act as a crucial buffer, mitigating the negative impact of depression on overall quality of life in patients undergoing maintenance hemodialysis [10]. Furthermore, the role of spiritual well-being has been identified as a significant factor, with studies indicating an inverse correlation between spiritual strength and symptoms of depression, anxiety, and stress in this patient population [11]. However, the complex interplay between resilience, spiritual well-being, gender, and dialysis duration within the unique socio-cultural landscape of the Saudi hemodialysis population is not yet understood. Therefore, this study was designed with the following objectives: (1) to quantify the prevalence of anxiety and depression among hemodialysis patients in Madinah, Saudi Arabia; (2) to assess their health-related quality of life (HRQoL) across multiple domains; (3) to analyze gender-based differences in these psychological parameters; and (4) to explore the correlation between dialysis vintage and psychological well-being. We hypothesize that the prevalence of anxiety and depression will be significantly high, with females reporting greater severity, and that longer dialysis vintage will correlate strongly with poorer psychological outcomes and lower HRQoL.

## 2. Materials and Methods

### 2.1. Study Design and Setting

A cross-sectional study was conducted from 15 January 2024, to 30 December 2024, at the dialysis units of multiple hemodialysis centres in Madinah, Saudi Arabia. This multi-center approach was adopted to enhance the representativeness of the sample and capture a broader spectrum of the patient experience within the region.

### 2.2. Participants

The study cohort comprised adult patients (≥18 years) who had been receiving maintenance hemodialysis for a minimum of three months, ensuring they were adapted to the dialysis routine. The exclusion criteria were deliberately structured to minimize confounding variables and included a diagnosis of severe cognitive impairment, as objectively assessed by a Mini-Mental State Examination (MMSE) score of <18; a documented history of active psychotic disorder; or an unwillingness to provide written informed consent. A total of 250 patients (130 males, 120 females) were ultimately enrolled via a consecutive sampling methodology, which helped reduce selection bias.

A formal sample size calculation was performed a priori using GPower software (version 3.1). The calculation was based on an anticipated medium effect size (w = 0.3) for the primary outcome of gender differences in HADS prevalence, drawn from preliminary data and consistent with previous literature [12]. With an alpha (α) level set at 0.05 and a desired statistical power (1 − β) of 0.80, the analysis indicated a minimum required sample size of 218 participants. The final enrollment of 250 patients provided an adequate buffer to account for potential incomplete responses and ensured robust statistical power for the planned subgroup analyses.

### 2.3. Data Collection

Data collection was executed through a structured process comprising two primary components:

*1.*  Demographic and Clinical Profile:
  A customized questionnaire was used to gather information on age, gender, dialysis vintage (recorded in years), key comorbidities (specifically hypertension and diabetes), and current employment status.
*2.*  Standardized Psychometric Instruments:
  Hospital Anxiety and Depression Scale (HADS): This 14-item self-report scale, with 7 items each for anxiety (HADS-A) and depression (HADS-D), is extensively validated for use in medically ill populations, as it minimizes confounding by physical symptoms [12]. In accordance with established guidelines, a subscale score of ≥8 was used as the cut-off point to indicate clinically significant symptoms of either anxiety or depression [13].

36-Item Short Form Health Survey (SF-36): This generic instrument was utilized to provide a comprehensive assessment of HRQoL across eight distinct domains: Physical Functioning, Role-Physical, Bodily Pain, General Health, Vitality, Social Functioning, Role-Emotional, and Mental Health [14]. To contextualize the degree of impairment in our cohort, the obtained SF-36 scores were compared to well-established US general population norms [15]. This methodological approach is standard in international HRQoL research, particularly when contemporary, localized population norms are unavailable, as it provides a consistent and validated benchmark against a large, healthy reference group.

### 2.4. Statistical Analysis

All statistical analyses were performed using IBM SPSS Statistics for Windows, Version 26.0. Descriptive statistics, including means, standard deviations, frequencies, and percentages, were computed to summarize the demographic and clinical characteristics of the participants. Group comparisons for categorical variables, specifically the prevalence of anxiety and depression by gender, were conducted using the Chi-square (χ^2^) test. For continuous variables, such as differences in SF-36 domain scores between male and female patients, independent samples *t*-tests were employed. To provide a more nuanced interpretation of the findings that extends beyond mere statistical significance, effect sizes were calculated and reported; Cramér’s V was used for chi-square tests and Cohen’s d for *t*-tests. The strength and direction of the linear relationships between dialysis duration (in years) and the continuous outcome variables (HADS scores, SF-36 domains) were analyzed using Pearson’s correlation coefficient (r). For all inferential analyses, a two-tailed *p*-value of <0.05 was deemed to denote statistical significance.

Note on Advanced Statistical Analysis: In response to a reviewer’s insightful suggestion, we explored the feasibility of performing more complex multivariate analyses, such as a two-way ANOVA, to examine the interaction effects between gender and age group on psychological outcomes. However, after detailed consultation and a review of our sample size distribution across the necessary demographic subgroups, it was determined that this specific analysis was not methodologically feasible. Proceeding would have risked creating underpowered cells with small counts, leading to potentially unreliable and spurious interpretations. Consequently, to maintain the statistical integrity and clarity of our findings, the analysis was prudently focused on the primary, pre-specified hypotheses concerning main effects, for which the study was explicitly designed and adequately powered.

## 3. Results

### 3.1. Demographics and Clinical Characteristics

The mean age of the participant cohort was 56.4 ± 12.3 years, with an age range from 24 to 82 years, indicating a representation across adult life stages. The mean duration of dialysis dependency, or vintage, was 3.8 ± 2.1 years, reflecting a population with substantial exposure to the treatment regimen. Hypertension and diabetes were the predominant comorbidities, affecting 68% (n = 170) and 52% (n = 130) of the participants, respectively. A striking 70% (n = 175) of the cohort were unemployed, with a statistically significant gender disparity observed; 78% of female patients were unemployed compared to 62% of males (*p* = 0.02). The full demographic breakdown is presented in Table 1.

### 3.2. Psychological Outcomes: Anxiety and Depression

The assessment using the HADS revealed a substantial burden of psychological morbidity. Overall, 38% (n = 95) of the patients scored at or above the clinical threshold for anxiety (HADS-A ≥ 8). A more granular analysis uncovered a significant gender disparity: 47% (n = 56) of females exhibited clinically significant anxiety compared to 30% (n = 39) of males (*p* = 0.01). Similarly, for depression, 32% (n = 80) of the total cohort scored ≥ 8 on the HADS-D subscale. Again, females were significantly more affected, with a prevalence of 40% (n = 48) compared to 25% (n = 32) in males (*p* = 0.02). To quantify the magnitude of these gender differences, effect sizes were calculated, yielding a Cramér’s V of 0.17 for anxiety and 0.15 for depression, both indicating small-to-medium practical significance. These results are summarized in Table 2.

### 3.3. Health-Related Quality of Life (HRQoL)

The SF-36 results demonstrated a severe and comprehensive impairment in HRQoL across all eight domains when compared to US general population norms. The deficits were dramatic and statistically significant (*p* < 0.001 for all domains). The Physical Component Summary was markedly low at 42.3 ± 9.1, compared to a norm of 72.5 ± 8.9. The Mental Component Summary was similarly compromised at 44.6 ± 10.2, versus a norm of 68.3 ± 7.4. A gender-based analysis further revealed that female patients reported statistically significant lower scores in specific domains, including Social Functioning (48.2 vs. 53.1; *p* = 0.03) and Emotional Well-Being (47.8 vs. 51.9; *p* = 0.04), suggesting a gendered dimension to the quality of life burden. The complete SF-36 scores are detailed in Table 3.

### 3.4. Correlational Analysis with Dialysis Vintage

Pearson’s correlation analysis yielded significant insights into the long-term impact of hemodialysis. Dialysis vintage, measured in years, demonstrated a significant positive correlation with worsening psychological scores: a modest but significant correlation with anxiety (r = 0.28; *p* < 0.001) and with depression (r = 0.24; *p* = 0.003). Concurrently, longer dialysis duration was strongly and negatively correlated with declining HRQoL, showing a moderate negative correlation with both the physical health domain (r = −0.34; *p* < 0.001) and the mental health domain (r = −0.29; *p* = 0.002) of the SF-36. These results are consolidated in Table 4.

## 4. Discussion

This comprehensive cross-sectional study provides a stark depiction of the severe psychological and quality of life burden endured by hemodialysis patients in the Madinah region of Saudi Arabia. The high prevalence of anxiety (38%) and depression (32%) aligns disquietingly with the upper ranges reported in the global literature [3,16], reinforcing the universality of this mental health crisis within dialysis populations. The pronounced gender disparity we observed, with females exhibiting a 1.5-fold higher risk for both anxiety and depression, is a critical finding that resonates with international trends [6] but demands a context-specific interpretation. In the Saudi socio-cultural context, this likely reflects the potent intersection of chronic illness with traditional female caregiving roles, potentially greater restrictions on social participation, and internalized stigma, which may amplify the stressors inherent to dialysis [17].

The profoundly low scores across all SF-36 domains quantitatively confirm that the impact of ESRD and its treatment is not confined to laboratory parameters but permeates every aspect of a patient’s existence. The dramatic deficit in physical functioning (42.3 vs. 72.5) translates to tangible realities such as an inability to perform basic household chores, while the crippling fatigue (Vitality: 47.1) stifles social engagement and employment, trapping patients in a cycle of isolation and dependency [8]. Our findings gain further clinical resonance when compared to historical data from other dialysis populations, such as the US-based study by Meyer et al. [18], which similarly documented severe HRQoL impairments, suggesting a universal “burden of therapy” that transcends geographical boundaries.

One of the most compelling findings of our study is the significant correlation between longer dialysis vintage and worse psychological and HRQoL outcomes. This suggests a “wear and tear” effect, where the cumulative stress, dietary monotony, and relentless cycle of treatment gradually deplete a patient’s physical reserves and psychological resilience over time [2,19]. This finding underscores the critical need for ongoing, rather than just initial, psychological support. Our results, viewed in conjunction with emerging research on psychological resilience [9,10], suggest that fostering resilience could be a key therapeutic target to interrupt this downward spiral. Similarly, the potential role of spiritual well-being as a coping resource, as indicated by Rezaei et al. [11], warrants serious consideration in future supportive care models, particularly in culturally rich settings like Saudi Arabia.

The evidence base for intervention is robust. A recent systematic review and meta-analysis by De Luca et al. [20] conclusively demonstrated that psychosocial interventions—encompassing cognitive-behavioral therapy (CBT), relaxation techniques, and structured psychoeducation—have a medium to large significant effect in reducing depression (SMD = −0.85) and anxiety (SMD = −0.99) in hemodialysis patients. Furthermore, physical interventions, such as intradialytic exercise programs, have shown promise not only in improving physical capacity but also in ameliorating psychological symptoms, as demonstrated in a recent pilot study [21]. Therefore, the integration of such non-pharmacological, multi-component interventions is not merely a recommendation but an ethical imperative for modern, holistic nephrology care.

## 5. Limitations

While this study offers valuable insights, its interpretation must be tempered by an acknowledgment of its limitations. The cross-sectional design, while efficient for determining prevalence, inherently precludes the establishment of causal relationships between dialysis vintage and psychological outcomes. The confinement of the study to a single geographical region within Saudi Arabia may limit the generalizability of the findings to other cultural or healthcare settings within the Kingdom or the wider Gulf region. Furthermore, the use of US-based population norms for the SF-36, while methodologically standard, introduces a potential cultural bias in the interpretation of HRQoL, as values and perceptions of health can vary significantly. As previously noted, constraints related to sample size distribution prevented the use of more complex multivariate models to explore intricate interaction effects, such as those between gender, age, and comorbidities. Finally, the study did not incorporate quantitative measures of potentially protective factors like resilience or spiritual well-being, which represents a fruitful avenue for future research. Future studies should employ longitudinal designs, include these psychosocial moderators, and aim for larger, nationally representative samples to enable more sophisticated analyses.

## 6. Conclusions

In conclusion, this research unequivocally demonstrates that patients undergoing hemodialysis in our study setting endure a severe and multifaceted burden, characterized by high rates of psychological morbidity and a drastically diminished quality of life, with women and long-term patients being particularly vulnerable. The findings argue compellingly for a paradigm shift in nephrology care—from a predominantly survival-focused model to a wellness-centered one that proactively addresses mental health. We strongly recommend the mandatory integration of routine, validated mental health screenings (e.g., HADS) into dialysis clinic workflows. Furthermore, based on the robust evidence, healthcare providers and policymakers should champion the development and implementation of tailored, gender-sensitive interventions. These could include structured support groups for women addressing caregiver burden, stigma-reduction programs for men, and the accessible provision of evidence-based therapies like CBT and intradialytic exercise. By systematically weaving psychological and social support into the fabric of renal care, we can transform dialysis units from sites of mere survival into therapeutic environments that foster holistic healing, resilience, and an improved quality of life for every patient.

## Figures and Tables

**Table 1:** Demographic and Clinical Characteristics of the Study Cohort (n = 250).

Variable	Total (n = 250)	Male (n = 130)	Female (n = 120)	*p*-Value
Age (years)	56.4 ± 12.3	55.8 ± 11.9	57.1 ± 12.8	0.41
Dialysis Duration (years)	3.8 ± 2.1	3.6 ± 1.9	4.0 ± 2.3	0.12
Hypertension	170 (68%)	85 (65%)	85 (71%)	0.31
Diabetes	130 (52%)	70 (54%)	60 (50%)	0.52
Unemployed	175 (70%)	80 (62%)	95 (78%)	0.02

**Table 2:** Prevalence of Anxiety and Depression Based on HADS Scores (N = 250).

Outcome	Total (n = 250)	Male (n = 130)	Female (n = 120)	*p*-Value
Anxiety (HADS-A ≥ 8)	95 (38%)	39 (30%)	56 (47%)	0.01
Depression (HADS-D ≥ 8)	80 (32%)	32 (25%)	48 (40%)	0.02

**Table 3:** SF-36 Quality of Life Scores (Mean ± SD) for Hemodialysis Patients vs. Population Norms.

Domain	Hemodialysis Patients	Population Norms	*p*-Value
Physical Functioning	42.3 ± 9.1	72.5 ± 8.9	<0.001
Role-Physical	38.7 ± 10.4	75.2 ± 9.3	<0.001
General Health	45.2 ± 8.7	68.9 ± 7.8	<0.001
Vitality	47.1 ± 9.5	66.3 ± 6.7	<0.001
Social Functioning	50.6 ± 11.2	73.8 ± 8.4	<0.001
Role-Emotional	44.6 ± 10.2	70.1 ± 7.9	<0.001
Mental Health	46.8 ± 9.8	69.5 ± 6.2	<0.001

**Table 4:** Correlation Coefficients between Dialysis Vintage and Key Outcome Variables.

Variable	r-Value	*p*-Value
Dialysis Duration vs. Anxiety	0.28	0.001
Dialysis Duration vs. Depression	0.24	0.003
Dialysis Duration vs. Physical HRQoL	−0.34	0.001
Dialysis Duration vs. Mental HRQoL	−0.29	0.002

## Data Availability

The data presented in this study are available on request from the corresponding author. The data are not publicly available due to privacy and ethical restrictions.
